# Smart Road Traffic Accidents Reduction Strategy Based on Intelligent Transportation Systems (TARS)

**DOI:** 10.3390/s18071983

**Published:** 2018-06-21

**Authors:** Abdulaziz Aldegheishem, Humera Yasmeen, Hafsa Maryam, Munam Ali Shah, Amjad Mehmood, Nabil Alrajeh, Houbing Song

**Affiliations:** 1 Traffic Safety Technologies Chair, Urban Planning Department, College of Architecture and Planning, King Saud University, Riyadh 11574, Saudi Arabia; 2 Department of Computer Science, COMSATS University Islamabad, Islamabad 45550, Pakistan; humera_9@ymail.com (H.Y.); hafsa.maryam09@gmail.com (H.M.); mshah@comsats.edu.pk (M.A.S.); 3 Institute of IT, Kohat University of Science & Technology, Kohat KP 26000, Pakistan; dramjad.mehmood@ieee.org; 4 Biomedical Technology Department, College of Applied Medical Sciences, King Saud University, Riyadh 11633, Saudi Arabia; nabil@ksu.edu.sa; 5 Department of Electrical, Computer, Software, and Systems Engineering, Embry-Riddle Aeronautical University, Daytona Beach, FL 32114, USA; h.song@ieee.org

**Keywords:** Intelligent Transportation System, accident, reduction, VANETs

## Abstract

Traffic accidents have become an important problem for governments, researchers and vehicle manufacturers over the last few decades. However, accidents are unfortunate and frequently occur on the road and cause death, damage to infrastructure, and health injuries. Therefore, there is a need to develop a protocol to avoid or prevent traffic accidents at the extreme level in order to reduce human loss. The aim of this research is to develop a new protocol, named as the Traffic Accidents Reduction Strategy (TARS), for Vehicular Ad-hoc NETworks (VANETs) to minimize the number of road accidents, decrease the death rate caused by road accidents, and for the successful deployment of the Intelligent Transportation System (ITS). We have run multiple simulations and the results showed that our proposed scheme has outperformed DBSR and POVRP routing protocols in terms of the Message Delivery Ratio (MDR), Message Loss Ratio (MLR), Average Delay, and Basic Safety Message.

## 1. Introduction

Over the past few decades, the number of vehicles has significantly increased which has resulted in populated roads. This massive traffic load has uplifted the road accident graph, which has consequently raised injuries and death rates worldwide. In 2010, the states presented in the World Health Report (WHR) documented road traffic injuries as the ninth most common reason for the disability of people. According to the report by the World Health Organization (WHO) [1], about 1.24 million casualties and 50 million injuries were reported globally. Automobile accidents are rated as the eighth cause of injuries and death toll worldwide. In the KSA, compared to other developed countries like the UK and USA, the death toll caused by road accidents has increased from seventeen percent to twenty-four percent per 10 million people over a decade [2] and is the primary reason for a young person’s death [3]. Friends of the Red Crescent Committee reported 526,000 accidents yearly in KSA with approximately seventeen casualties per day. According to the report published in the Aleqtisadiah newspaper, there were 2.8% more car accidents in 2016 than those reported in 2015. On the basis of these statistics, it is estimated that the death toll will further grow by 2020 due to road accidents [4,5]. Attention must be given towards this severe road traffic condition in the KSA to control this, otherwise it will probably increase to four million crashes per year by 2030 according to the prediction made in [6].

These road traffic accidents not only cause an increased death toll or injury, but also result in communal and financial damage to the state [7]. The deaths or disabilities of youngsters caused by accidents have a solemn effect on families and more widely on society [8]. The loss or disability of an earner not only causes financial suffering for family members, but also changes the domestic dynamics. On the other hand, the state expends SR 13 billion approximately per year to matters associated with road accidents [9]. Therefore, it is essential to find a way to avoid such severe conditions so that the state can continue to prosper in terms of productivity, as well as socially and economically.

Numerous efforts have been made to avoid such drastic situations through the implications of speed limits, imposition of traffic rules, deployment of seat belts and air bags, boosting stiffness into the physical structure of a vehicle, and so on [10]. Furthermore, researchers have directed their attention to avoiding accidents and have introduced a number of active electronic and computer-controlled mechanisms such as braking systems, intelligent speed adaptation, collision prevention, self-governing cruise control systems, electronic stability control systems, and so on [11]. In recent times, Intelligent Transportation Systems (ITS) have gained much attention from the research community [12]. ITS introduced smart vehicles that are enabled to wirelessly communicate with one another through a communication device called an On-Board Unit (OBU). A network of inter-connected vehicles equipped with Electronic Control Modules (ECMs) are called Vehicular Ad-hoc NETworks (VANETs) [13].

Vehicular Networks are the most important and emerging technology in the field of Intelligent Transportation Systems (ITS). A large number of new routing schemes and architectures have been suggested in recent years in order to successfully deploy ITS. The widespread deployment of ITS is a complex and challenging task. Numerous projects have been developed and their performance evaluated either through simulation or deployment in a real environment. Various projects developed by different countries include the project related with Automobile Safety Communication in the United State of America (USA) and Car-to-Car communication in the European Union. The European Union, USA, and Japan have already deployed a smart vehicular environment in both urban and highway areas. Many car manufacturing companies like Ford, Daimler, BMW, General Motors, and Audi have been inspired by the ITS and are motivated to manufacture smart vehicles for passenger safety.

In VANETs, the topology of the network keeps changing due to the high mobility of vehicles, so the network is considered as an ad-hoc network. It enables vehicles to sense their environment and exchange their sensed data with surrounding vehicles. An infrastructure named Road Side Units (RSU) is installed along the roads to assist the vehicles moving in its vicinity. Standard IEEE 802.11p [14] has been introduced, particularly for vehicular communication, which allows ad-hoc communication (the *p* denotes the specific version for communication between vehicles). There are three modes of communication in VANETs: Vehicle to Infrastructure (V2I), Vehicle to Vehicle (V2V), and Vehicle 2 Hybrid (V2X) communication. Vehicles communicate with each other in order to get a better understanding of the surrounding environment to prevent any hazardous situations. Drivers must be given timely warnings about any expected hazardous situations in order to avoid accidents. Another concern is that drivers become doubtful as to which route to follow next even if warned about emergency situations, which can cause inefficient traffic flow and congestion on alternate routes [15]. Traffic safety and smooth flow can be achieved by assisting the driver with appropriate suggestions. The aim of this project was to design and develop a possible accident detection strategy that provides timely warnings to the driver about possible accident situations and gives appropriate suggestions accordingly.

The objectives of this research were focused on the following aspects:

(1) The design of a new protocol for accident prevention and, hence, reduction.
(2) To forecast/guess the probability of the occurrence of an accident in advance before it occurs.
(3) To re-route vehicle traffic to prevent traffic jams on the road that may cause accidents.
(4) To maintain traffic flow efficiently.
(5) To assist drivers to reach a destination on time.
(6) To reduce safety messages broadcasting in order to avoid a broadcasting storm and network congestion.
(7) To minimize the delay in re-routing of traffic to other available paths.

The roadmap of this research is arranged as follows. An overview of the existing protocols proposed to avoid traffic accidents is presented in Section 2. In Section 3, a smart road traffic accident reduction strategy is presented in detail. The simulation parameters of the proposed strategy are presented in Section 4. The simulation results of the proposed scheme are discussed and evaluated in Section 5. Finally, the research work conclusions are presented in Section 6.

## 2. Literature Review

**Figure 1:**
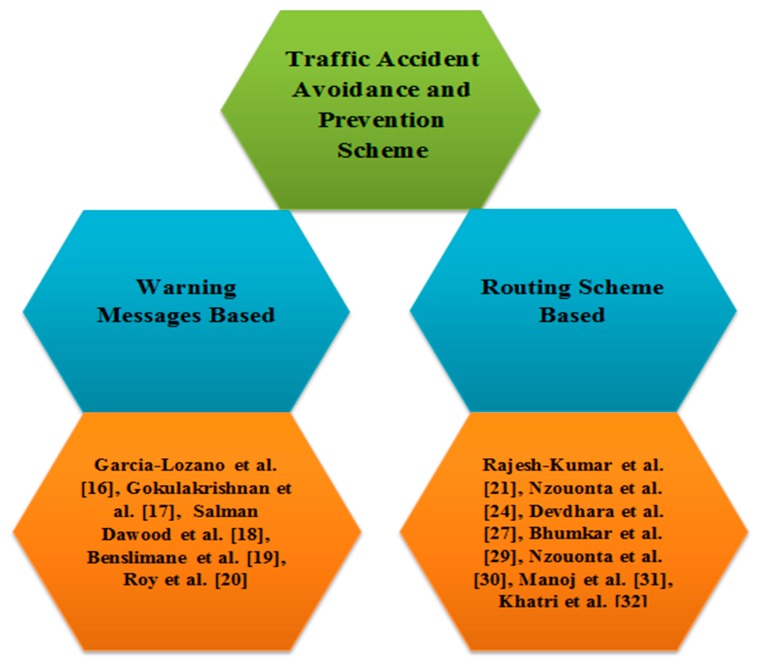
Taxonomy of Literature Review.

This section describes a list of the extensive research conducted to avoid road accidents at the maximum level with a specific focus on the vehicular environment in order to reduce human loss. The Literature Review section is further divided into two sections for each category of traffic accident avoidance and prevention schemes, based on routing schemes and warning messages as shown in Figure 1.

### 2.1. Traffic Accident Avoidance and Prevention Based on Warning Messages

Lozano et al. [16] proposed the warning message scheme to avoid traffic accidents between vehicles by sending a warning message to alert drivers about the condition of the current accident. The proposed scheme used the distance-based flooding scheme. The author calculated the time of the vehicle’s reaction after the occurrence of the accident in order to prevent further traffic accidents. Furthermore, a warning message dissemination scheme has been introduced for low priority messages in order to achieve efficient bandwidth utilization. The proposed routing scheme achieved the best high and low priority message dissemination in drastic weather conditions, i.e., rain, sun, etc. Furthermore, the routing scheme achieved less delay and an efficient utilization of the bandwidth under different traffic conditions. However, the limitation of this routing scheme is that it does not work in a highly dense vehicular environment. Another limitation of this scheme is the highest priority message that the author used in the simple flooding scheme. So, the time utilized in taking the forwarding decision was less. Therefore, the proposed scheme faces scalability issues related to the dissemination of high priority messages.

Gokulakrishnan et al. [17] proposed an accident avoidance routing scheme named Road Accident Prevention (RAP). This scheme introduced the Early Warning (EW) message in order to make essential decisions—selecting alternate routes, slowing down the vehicle, and changing lanes. Furthermore, the Road Side Unit (RSU) detects any unusual activity in the highway scenario and broadcasts an Early Warning (EW) message to all the vehicles in the range of the RSU. In this way, the EW message must be sent at the exact time to prevent the road accident. The authors introduced different types of risk zone, namely, high, average, and low risk zones. The high-risk zone comprises those vehicles that are nearest to vehicles that are involved in road accidents. The routing scheme successfully achieved less delay. However, the limitation of the proposed scheme was the high network processing overhead due to the extra warning message transmitted, the EW message and identification of the risk zone.

Dawood et al. [18] proposed an accident avoidance scheme named the Efficient Emergency Message Broadcasting (EEMB) routing scheme. The objective of the proposed scheme was to reduce traffic bottlenecks and prevent multiple road accidents by broadcasting emergency messages with low overheads at high velocity. After an accident has occurred between two vehicles, the affected vehicle will select the best forwarder and broadcast the emergency message until the emergency message covers the whole risk zone. The forwarding scheme achieved the minimal overhead caused by the beacon message by using the method of the early prediction system. The beauty of this forwarding scheme is that it overcomes the problem of network fragmentation by using the mechanisms of store-carry and forward.

Benslimance et al. [19] considered that the consumer with the emergency message must disseminate the message intermittently until it selects another forwarder vehicle. However, this dissemination causes a duplication of the broadcasting message due to multiple broadcasting attempts that do not help the vehicle to obtain another vehicle. Therefore, the network of the traffic will be affected from the processing overhead. In order to minimize the processing overhead, the author proposed a scheme named Optimized Dissemination of Alarm Message (ODAM) while restricting the regions and forwarder vehicles. The ODAM routing scheme efficiently achieves less overhead due to the periodical broadcasting message. However, the ODAM scheme experiences a high delay by sending the emergency messages to the forwarder vehicle.

Roy et al. [20] proposed accident avoidance and congestion prevention in a vehicular environment. In this scheme, after the accident occurs, the affected vehicle generates the emergency message. The RSU receives the emergency message and then retransmits a further emergency message to the other RSUs which lie in its range. The beauty of this routing scheme is that it prevents the duplication of an emergency message. Before receiving the emergency message, the RSU will check whether the same emergency message has been received or not. In the case of not being received, the RSU will accept the emergency message, otherwise not. This routing scheme has successfully achieved less delay, a high delivery ratio, and maximum throughput.

### 2.2. Traffic Accident Avoidance and Prevention Based on Routing Scheme

Kumar et al. [21] proposed a Distance-Based routing scheme. The basic idea behind this routing scheme is to avoid the accident at the intersection as, most of the time, anonymous vehicles appear from the other side of the road and cause accidents at the intersection. This scheme for accident prevention starts by obtaining the vehicle location and calculating the distance of the vehicles near to the intersection. After calculating the location of each vehicle, all vehicles will obtain information on the location of other vehicles coming towards the intersection. As a result, the vehicle with the shortest distance will send an alert to other vehicles about its distance and location. The proposed scheme successfully achieved less traffic congestion, which helps to prevent or reduce traffic accidents. However, the scope of this proposed routing scheme was limited and faces scalability issues. Moreover, it only works for an intersection, where three different roads meet/join at a single location, and a ring road.

Kshirsagar et al. [22,23] proposed an intelligent vehicular management scheme to prevent traffic accidents. The proposed scheme used basic warning safety messages to prevent traffic congestion. The traffic signal and management scheme successfully achieved high throughput, a high delivery ratio, and less delay.

Nzouonta et al. [24] proposed a scheme named Spatio-Temporal Emergency Information Dissemination (STEID). The goal of this routing scheme is to suggest a hybrid network architecture by using cellular links and proxy servers. The STEID routing protocol achieved a maximum ratio of successfully delivered alert emergency messages, less traffic load, and fewer delays related to the message reception rate. However, the STEID routing scheme is costly as a large-scale infrastructure is needed in order to cover a long path. Furthermore, the hybrid network architecture does not match the requirements related to traffic safety applications. This generally requires V2V communication to be efficient for delivering the data at the lowest cost [25,26].

Devdhara et al. [27] considered a traffic avoidance routing protocol named Inter-Vehicle Collision (IVC). All vehicles in the cluster broadcast a secure warning message to give further information to the other vehicles, for example, a traffic jam [28]. This scheme only detects collisions when the vehicle changes its path. In order to ensure IVC performance, the author used methods such as K-Mean Clustering and Agglomerative Hierarchical Clustering (AHC) [28]. The AHC algorithms had an outstanding performance when compared to K-Mean Clustering in order to detect accidents that lie in the identical cluster as well as to send data about the accident to those vehicles which lie in the cluster.

Bhumkar et al. [29] proposed a driver fatigue detection scheme by using real time sensors. In the proposed scheme, the driver’s fatigue will be detected immediately; if the driver is found to have drunk alcohol, they are warned through a notification message and the ignition is turned off. In this way, the possibility of traffic accidents is avoided. The fatigue detection scheme has an outstanding performance in human behavior detection in vehicles.

Nzouonta et al. [30] considered a routing scheme named Road Based Vehicular Traffic (RBVT). The RBVT scheme uses real time information based on the vehicular environment to create a road-based intersection with network connectivity and high probability among vehicles. The proposed routing scheme uses the geographical forwarding scheme to transmit interest packets between road intersections on the route. The RBVT achieved the average packet delivery ratio and average delay due to its traffic overhead.

Manoj et al. [31] proposed a congestion detection algorithm to avoid traffic accidents caused by traffic congestion. After detecting traffic congestion, the drivers of the vehicles provide multiple options about the magnitude and location to avoid getting stuck in the traffic congestion. In Vehicle 2 Infrastructure (V2I) communication, after detecting traffic congestion in the lane due to a traffic accident, the affected vehicle, which is involved in the traffic accident, broadcasts the warning message to the other forwarder vehicle and RSU to inform them of the present condition of the lane. In this way, the next upcoming vehicle changes their decision to avoid traffic congestion and road accidents. This scheme achieved efficient bandwidth utilization and minimum message overhead.

Khatri et al. [32] proposed a traffic congestion detection routing scheme to avoid traffic accidents. The beauty of this proposed scheme is that it depends on the data collection and central network infrastructure. In data collision, data are gathered from the real-time environment. Vehicles are equipped with GPS to communicate with other vehicles. Collected data only become beneficial when it is shared with other vehicles, including velocity and present location. When any vehicle broadcasts the message to the path, the forwarder vehicle collects the congestion message and then checks whether the area is congested or not. If not, then the vehicle stores this message in its own memory. Otherwise, it simply shares the congestion location with the other vehicles. The proposed scheme achieved less transmission overhead and efficient utilization of the bandwidth. However, this scheme only worked in a homogenous vehicular environment, but was not tested in a heterogeneous vehicular environment.

### 2.3. Discussion

In this section, we discussed the traffic accident prevention and avoidance scheme with a specific focus on VANETs. We evaluated research papers from 2011–2017. In Table 1 and Table 2, we provide a concise and detailed review of different road accident and prevention schemes based on warning messages and routing schemes. It can be observed that road congestion and traffic accidents cause big trouble after an accident happens. In the accident area, vehicles get congested due to a lack of awareness about the road accident. Furthermore, vehicles coming from the road intersections cause traffic jamming. The scenario affects the routine of human life. To address the above problems there is a need to develop a routing protocol in order to avoid the road accidents as well as traffic congestion in VANETs’ environment. Furthermore, the protocol must warn the vehicle drivers promptly about any expected hazardous situations in order to avoid accidents. The protocol also gives an alternate route to drivers in order to follow the next shortest path, even if warned about the emergency situation, to overcome the traffic congestion situation, otherwise inefficient traffic flow and congestion at different routes will result [15].

## 3. Smart Road Traffic Accidents Reduction Strategy

In this section, we propose a strategy named as the Traffic Accidents Reduction Strategy (TARS) to avoid accidents and to prevent congestion in VANETs. In the proposed strategy, the warning message is transmitted in time; therefore, potential vehicle collisions are avoided. Moreover, vehicles are assisted to another route. In this section, first, an introduction to some of the participating entities is presented. Second, a few assumptions were made for the smooth execution of our proposed strategy. Then, phases of Traffic Accidents Reduction Strategy (TARS) are described in detail. Following are the entities involved in the execution of the TARS.

### 3.1. Entities

The entities involved in the traffic accident reduction strategy are as follows:

(1) Smart Vehicles
(2) Road Side Units (RSUs)
(3) Trusted Authority (TA)
(4) Governmental Authority (GA).

(1) *Smart Vehicles*

In VANETs, a communicating device called the On-Board-Unit (OBU) is installed in each vehicle which allows it to communicate with other entities in the system. Vehicles communicate with other vehicles and the RSU through the OBU to obtain a better awareness of their surroundings in order to react in a timely manner to any unusual situation, for example, accident, traffic jam, road blockage, and so on.

(2) *Road Side Units (RSUs)*

The RSU keeps the information of the automobiles (vehicles) moving in its transmission range so that it has a global view of the traffic. This feature enables the RSU to maintain the efficient traffic flow and, hence, accidents are also avoided.

(3) *Trusted Authority (TA)*

The Trusted Authority (TA) is a trusted administrative authority. Both vehicles and RSUs are registered with the TA. The TA assigns unique identities to both the RSUs and vehicles. The TA has a record of all the registered entities so that they remain liable to it.

(4) *Governmental Authority (GA)*

The Governmental Authority (GA) plays the role of a passive entity. The passive entity is not directly involved in the execution of TARS; however, it plays a vital role if a non-registered vehicle is encountered so that it should not affect the security of the VANET system at all.

### 3.2. Assumptions

For the smooth and efficient flow of our proposed strategy, we made the following assumptions:

1. All vehicles must be equipped or armed with a Global Positioning System (GPS) in order to obtain the exact location so that it can be used in the Basic Safety Message (BSM).
2. The RSU is an uncompromised entity. An uncompromised entity means that it can neither be effected by any attack nor can its record can be modified.
3. All vehicles and RSUs are registered with the TA.
4. The Vehicle to Infrastructure (V2I) communication mode is considered.

### 3.3. Phases of Traffic Accidents Reduction Strategy (TARS)

The Traffic Accidents Reduction Strategy (TARS) is divided into three phases. The first is the setup phase, which describes the process of establishing the VANET according to our proposed strategy; the authorization phase, the second phase of TARS, demonstrates the registration process of the entities. Finally, the execution of the proposed strategy is presented in the execution phase. We discuss these three phases in detail.

(1) *Setup Phase*

In this phase, the entities of VANETs are set up for the TARS scheme. A RSU periodically broadcasts its identity and location in its vicinity so that vehicles moving in its range can communicate with it. A vehicle broadcasts BSM containing VID, location, speed and direction to the RSU when it enters into the range of RSU and listens to RSU’s beacons. The RSU keeps the information of the automobiles (vehicles) travelling in its vicinity through the information received in the BSMs. The RSU data include the vehicle-ID, their velocity (V), location, and Tour-Span (TS). Tour-Span is the minimum time a vehicle spends in the range of a RSU. On reception of the BSM, the RSU extracts the vehicle-ID and verifies it by looking it up in its database. After verifying the vehicle-ID, the RSU calculates the tour-span ‘TS’ of the vehicle in its region as shown in Equation (1).

(1) $${\mathrm{TS}}_{i}=\frac{{Flocation}_{RSU}-{Position}_{i}}{{Velocity}_{i}}$$

where *Flocation_RSU_* is the end point of the transmission range of the RSU; *Position_i_* is the current location of a vehicle received in the BSM; and *Velocity_i_* is the velocity of vehicle *i.*

(2) *Authorization Phase*

**Figure 2:**
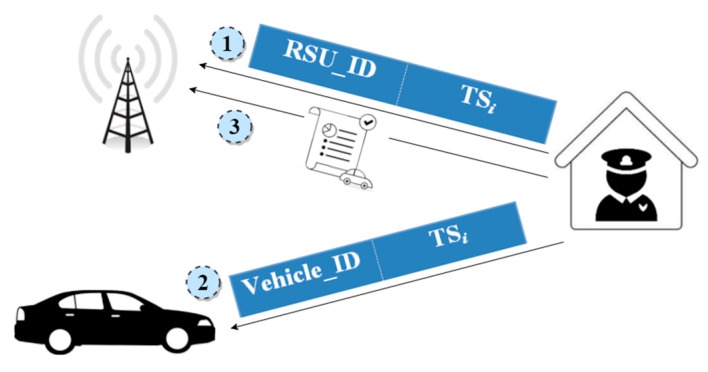
Authorization Phase.

This phase describes the procedure of registering the RSUs and vehicles with a Trusted Authority (TA). The TA is a trusted administrative authority that is responsible for the accountability of entities of the VANET system. Once the entities, RSUs, and vehicles become registered with the TA, then they remain liable to it. During the registration process, the TA provides a key and a vehicle ID to a vehicle as shown in Figure 2. A vehicle owner must keep this key in a safe place, for example, at home or at the bank, so that the owner can claim ownership of a vehicle if a vehicle becomes physically compromised. Then, the vehicle credentials can be revoked and any possible attacks, for example, misleading attack, can be avoided. The TA hands out the identities of registered vehicles to the RSUs installed at that region as people often travel in the region they live in. A question may arise as to what will happen if a vehicle registered in another region arrives/enters into the transmission range of a RSU. For this purpose, we present different scenarios in the next subsection.

(3) *Execution Phase*

**Figure 3:**
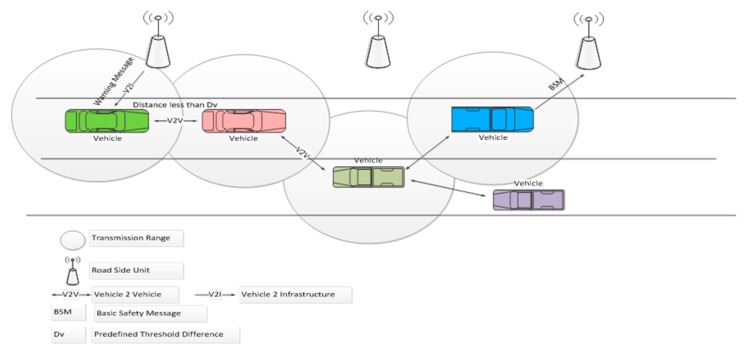
Illustration of Traffic Accidents Reduction Strategy (TARS).

The TARS focuses on the use of the distance measurement approach for warning message generation to prevent accidents along with the efficient rerouting of traffic. An illustration of the proposed technique is shown in Figure 3. The RSU broadcasts the beacon message containing its identity and location in its range so that vehicles moving in its vicinity can communicate with it. The vehicle broadcasts the Basic Safety Message (BSM) to the RSU when it listens to the beacon message of a RSU. The BSM consists of the vehicle id (Vehicle-ID), location, speed, and direction. The RSU maintains a map between the vehicle identity and location of each vehicle moving in its range. The RSU adds the information about a vehicle according to the latest BSM received from a vehicle. Vehicles then broadcast the BSM every *t* milliseconds. The RSU updates the record at the reception of each BSM. If a distance between the two vehicles, moving on same road lane, is less than the safe distance (*D_v_*) then the leading vehicle sends warning message to the following vehicle. On the other hand, the RSU also monitors the distance (D) and velocity (Velocity*_i_*) of each vehicle. It sends a warning message to warn that vehicle. *D_v_* is calculated using the method presented in Section 3.4. Whenever the distance between the two vehicles is less than *D_v_*, the RSU sends a warning message to the following vehicle to keep the safe distance. If in a certain time period (*T_p_*), the RSU has sent *T_alert_* messages, then it means there is a high traffic load in that area. The values considered for *T_p_* and *T_alert_* were 10 s and 4, respectively. These values were taken from [34]. Afterwards, the RSU sends a high traffic congestion message to the previous neighboring RSU to reroute the traffic. The neighboring RSU will reroute the upcoming vehicles as shown in Algorithm 1. In case vehicles are moving outside the range of the RSU, and the distance between the two vehicles becomes less than *D_v_* at some time, the leading vehicle sends a warning message to the following vehicle to keep a safe distance.

Meanwhile, it also reports it to the previously connected RSU through neighboring vehicles. Neighboring vehicles only act as relay nodes and they pass on the message to the trailing vehicles until it reaches that particular RSU.

**Algorithm 1:** Traffic Congestion Avoidance Algorithm

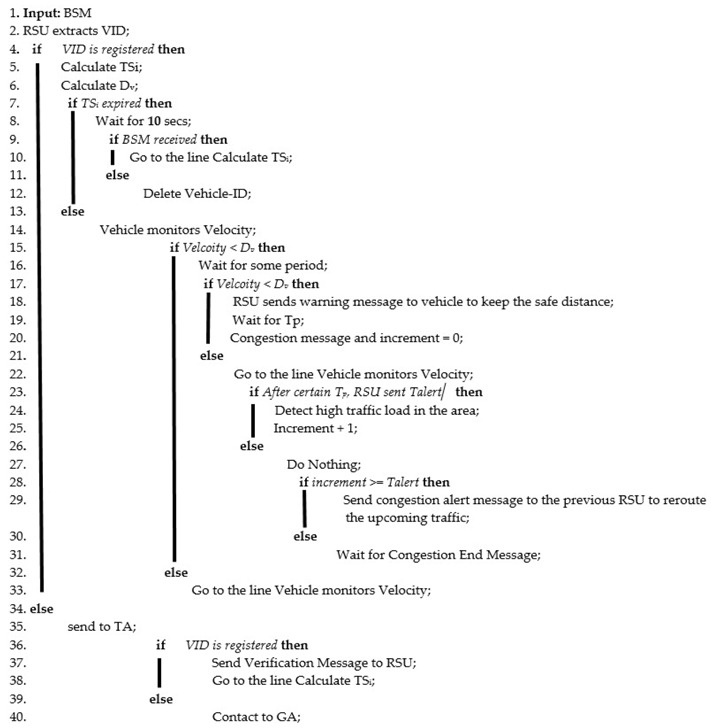



**Figure 4:**
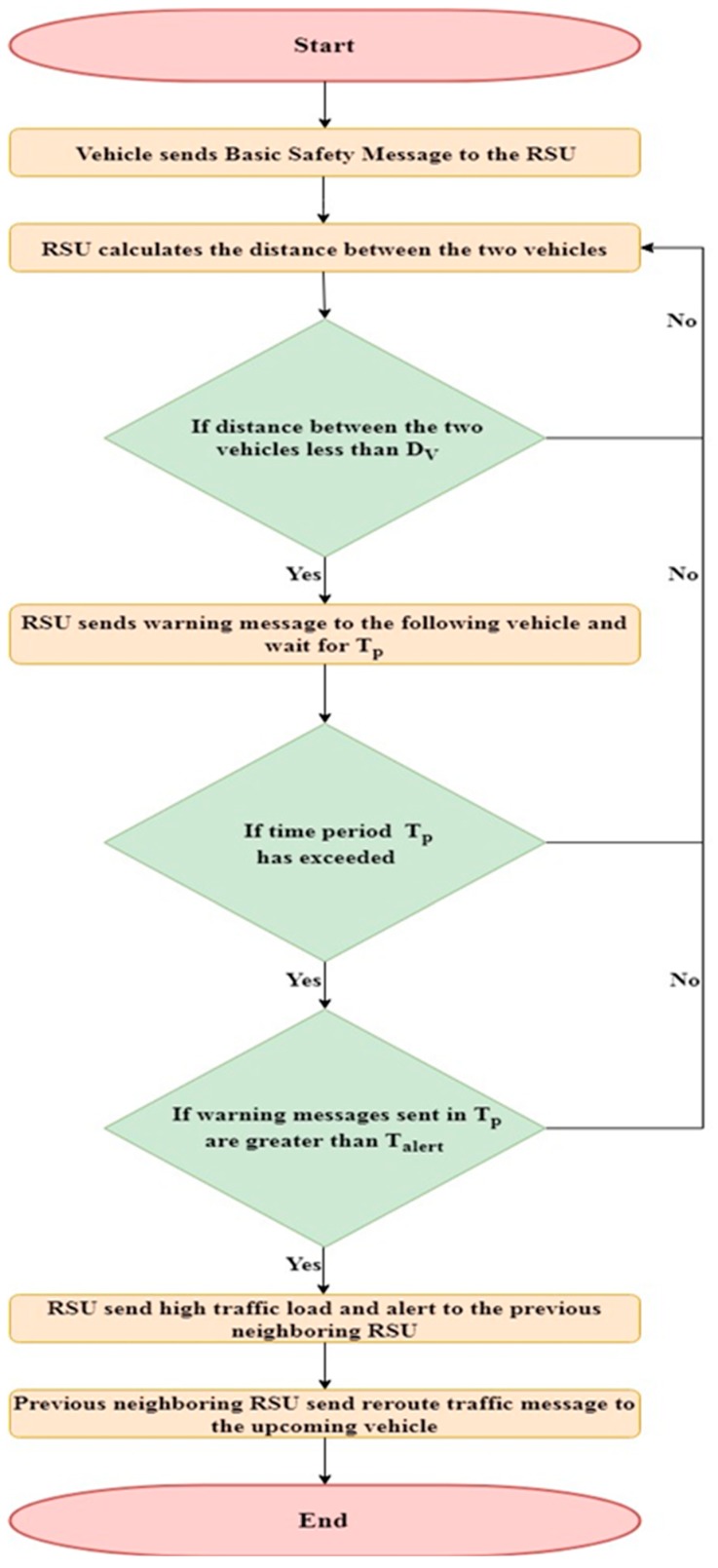
Workflow of TARS.

A flowchart of the methodology of the proposed strategy is shown in Figure 4.

**Scenario 1:**

**Figure 5:**
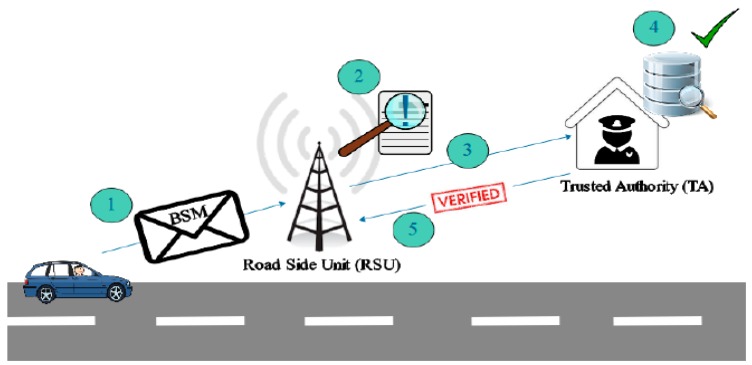
Scenario 1 illustration.

If a vehicle travels outside of the region in which it is registered, then the RSU of the newly entered region contacts the TA for the verification of a vehicle. The TA checks whether the vehicle is a registered with it or not. The TA then sends a verification message to the RSU if it is a registered vehicle as illustrated in Figure 5.

**Scenario 2:**

**Figure 6:**
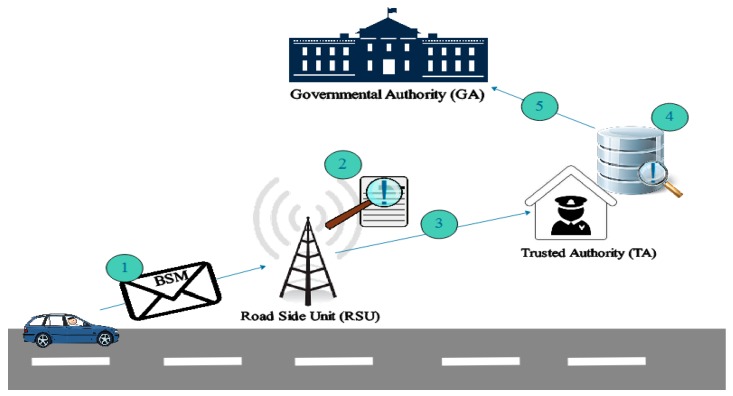
Scenario 2 illustration.

If a non-registered vehicle enters into the range of a RSU, it then contacts the TA for vehicle verification. The TA reports it to the Governmental Authorities (GA) for further action if that vehicle is not registered at the TA as shown in Figure 6.

### 3.4. Safety Distance Calculation Method

Let *a* and *b* represents the following and leading vehicle. The change in position of a leading vehicle w.r.t. the following vehicle can be calculated as shown in Equation (2):

(2) $$\Delta_{\left(a,b\right)}=\frac{{\left(v_{a}-v_{rel\left(a,b\right)}\right)}^{2}}{2\;declr_{m}}$$

where $declr_{m}$ is the maximum declaration allowed in the system and $v_{rel\left(a,b\right)}$ is the relative velocity between vehicle a and b which can be calculated as shown in Equation (3):

(3) $$v_{rel\left(a,b\right)}=v_{b}-v_{a}$$

The change in position of a following vehicle can be calculated as shown in Equation (4):

(4) $$\Delta_{\left(a\right)}=\frac{v_{a}{}^{2}}{2\;declr_{m}}$$

Safety distance is denoted by $S.D_{\left(a,b\right)}$ and it is difference of a change in position of following and leading vehicles as illustrated in Equation (5).

(5) $$S.D_{\left(a,b\right)}=\Delta_{\left(a\right)}-\Delta_{\left(a,b\right)}$$

By putting all the values $\Delta_{\left(a\right)}-\Delta_{\left(a,b\right)}$ in Equation (5), it can be simplified as shown in Equations (6)–(11):

(6) $$S.D_{\left(a,b\right)}=\frac{v_{a}{}^{2}}{2\;declr_{m}}-\left[\frac{{\left(v_{a}-v_{rel\left(a,b\right)}\right)}^{2}}{2\;declr_{m}}\right]$$

(7) $$S.D_{\left(a,b\right)}=\frac{v_{a}{}^{2}}{2\;declr_{m}}-\left[\frac{v_{a}{}^{2}+v_{rel\left(a,b\right)}{}^{2}-2\;v_{a}v_{rel\left(a,b\right)}}{2\;declr_{m}}\right]$$

(8) $$S.D_{\left(a,b\right)}=\frac{v_{a}{}^{2}}{2\;declr_{m}}-\frac{v_{a}{}^{2}-v_{rel\left(a,b\right)}{}^{2}+2\;v_{a}v_{rel\left(a,b\right)}}{2\;declr_{m}}$$

(9) $$S.D_{\left(a,b\right)}=\frac{v_{a}{}^{2}-v_{a}{}^{2}-v_{rel\left(a,b\right)}{}^{2}+2\;v_{a}v_{rel\left(a,b\right)}}{2\;declr_{m}}$$

(10) $$S.D_{\left(a,b\right)}=\frac{-v_{rel\left(a,b\right)}{}^{2}+2\;v_{a}v_{rel\left(a,b\right)}}{2\;declr_{m}}$$

(11) $$S.D_{\left(a,b\right)}=\frac{v_{rel\left(a,b\right)}\left[2\;v_{a}-v_{rel\left(a,b\right)}\right]}{2\;declr_{m}}$$

## 4. Performance Evaluation

**Figure 7:**
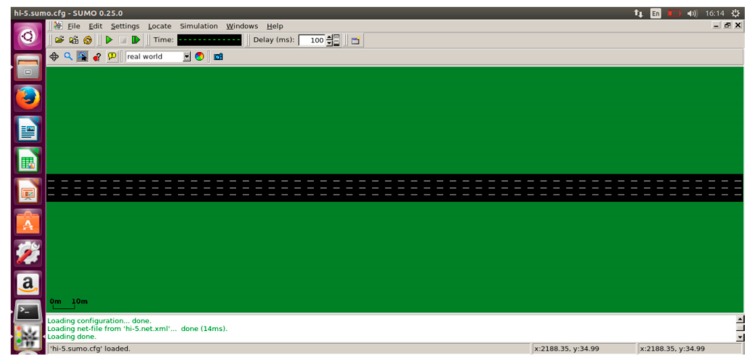
Highway Scenario using SUMO.

**Figure 8:**
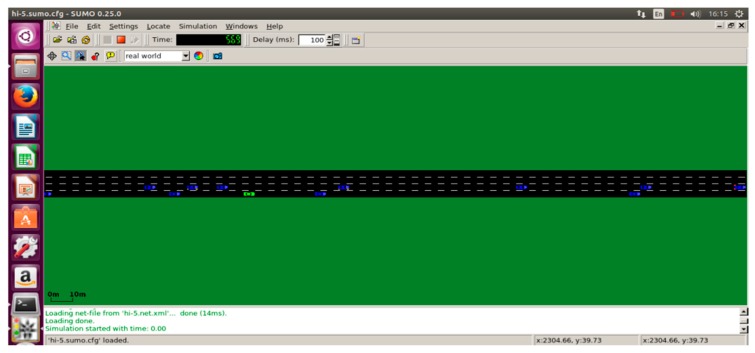
Real time traffic on highway using SUMO.

The performance of the proposed TARS routing protocol is analyzed and evaluated by using a real traffic simulation tool called the Simulation of Urban Mobility (SUMO) [35] integrated with the NS-2.35 [36]. The TARS protocol simulation is implemented in a 1000 × 1000 m^2^ grid map with a different number of vehicular nodes. All vehicular nodes were deployed randomly with a randomly generated velocity range from 60 km/h to 120 km/h. Vehicles are uniformly distributed over four lanes of a road as shown in Figure 7 and Figure 8.

The TARS protocol simulation parameters are shown in Table 3.

### 4.1. Simulation Tool of the TARS Routing Protocol

#### 4.1.1. Simulation of Urban Mobility (SUMO)

The SUMO tool is used to simulate the real vehicular traffic. In order to implement a realistic highway scenario, we obtained the mobility file of the real time traffic using SUMO. A screenshot of a real highway environment using SUMO is shown in Figure 8.

#### 4.1.2. Network Simulator (NS-2.35)

**Figure 9:**
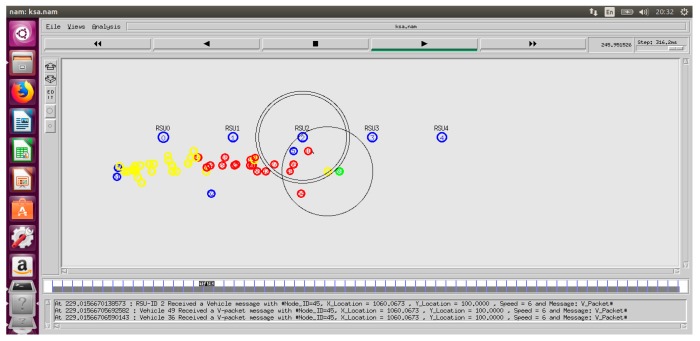
Simulation of the TARS protocol.

NS-2.35 is another tool used in the TARS routing scheme to implement the main purpose/procedure of the TARS routing protocol as shown in Figure 9.

**Figure 10:**
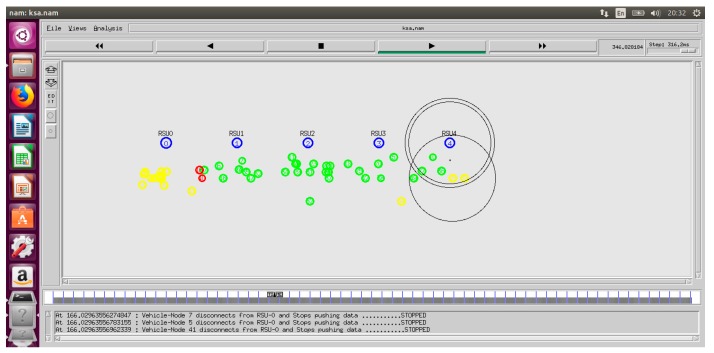
TARS simulation by using NS-2.35.

The mobility trace file is imported in NS-2.35. The TARS protocol simulation is presented in NS-2.35 as shown in Figure 10.

We focused on the highway scenario for the TARS simulation. The TARS scenario was implemented in a VANETS environment by using NS-2 and SUMO. We ran the simulation twenty-five times for each vehicle density parameter, and average results are shown in graphs.

### 4.2. Performance Evaluation Metrics

The TARS, DBSR [37], and POVRP [38] routing schemes were evaluated using a highway scenario in VANETs’ environment on the basis of the following performance parameters: Message Delivery Ratio (MDR), Message Loss Ratio (MLR), Average Delay, and Basic Safety Message (BSM). All of the above parameters are discussed in detail as follows.

#### 4.2.1. Message Delivery Ratio (MDR)

The MDR shows the percentage of messages that are successfully delivered to the vehicles. The basic objective of each routing protocol is to gain a high delivery ratio. The following mathematical formula is used to calculate the MDR.

MDR = (Total No. of Received Messages/Total No. of Sent Messages) × 100

#### 4.2.2. Message Loss Ratio (MLR)

The MLR shows the percentage of lost packets that are dropped during the transmission. The following formula is used to calculate the MLR.

MLR = 100 − (Total No. of Received Messages/Total No. of Sent Messages) × 100

#### 4.2.3. Average Delay

The Average Delay is the time taken by the RSU to send the safety warning message to the vehicle when two vehicles cannot maintain the predefined threshold distance between each other.

#### 4.2.4. Basic Safety Message (BSM)

The Basic Safety Message (BSM) shows the number of messages transmits from RSU to the vehicle or vehicle to vehicle.

## 5. Results and Discussion

Now, we discuss the experimental results of our proposed protocol, TARS, and compare it with the results obtained for DBSR and POVRP protocols using the highway scenario on the basis of the performance parameters which are discussed in the previous section.

### 5.1. Basic Safety Message (BSM)

**Figure 11:**
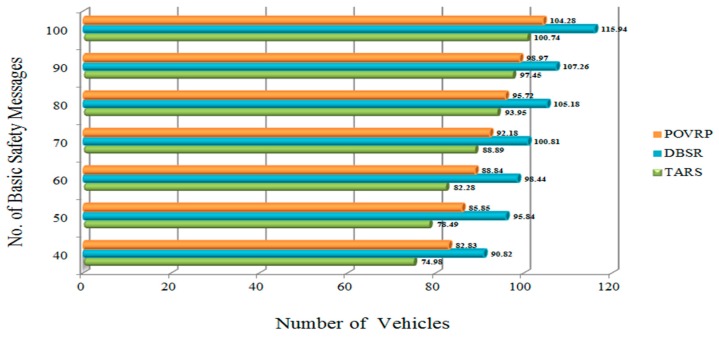
Basic Safety Message in the highway scenario.

The BSM plays a very important role in VANETs’ environment to transfer safety messages. Safety messages need to be transmitted all the time to all neighbor vehicles. In order to provide reliability in the traffic network, safety messages must reach their destinations without causing channel congestion. Furthermore, sending safety messages without using a congestion control or avoidance mechanism causes the broadcasting storm problem. In TARS protocol, when the number of vehicles increases, then the number of basic safety messages also increases. Moreover, RSU makes intelligent decisions, sending safety messages to the other vehicles while considering the dynamic nature of topology and the broadcasting storm problem in order to adjust, and considers the current road traffic information. The RSU monitors the distance between two vehicles, if the distance is less than the defined threshold, then the RSU sends warning messages in order to make the vehicle aware of the status of the traffic network (whether it is congested or not) and distance. In order to provide the stability of the channel network, the RSU and vehicle only send warning and safety messages when it is really needed. In TARS, the number of basic safety messages are fewer compared to DBSR and POVRP, meaning TARS decreases the chances of the occurrence of channel congestion in order to provide the stability of the network protocol as well as improving the network performance depending on the capacity of the channel. The RSU sends warning messages after verifying the possibility of congestion occurrence in terms of high traffic load in the network, and informs the upcoming vehicle to take alternate routes to avoid congestion as shown in Figure 11.

### 5.2. Average Delay

**Figure 12:**
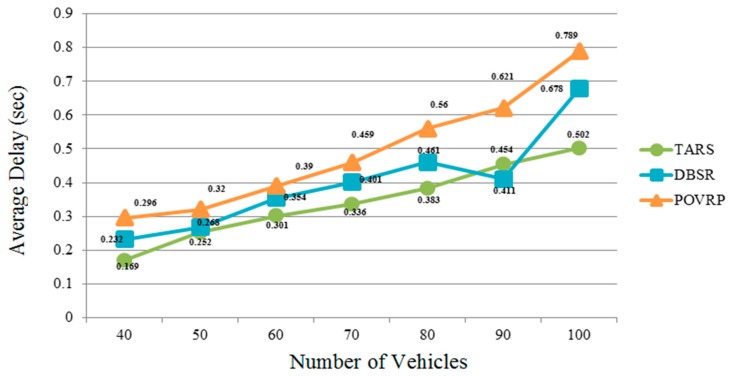
Average Delay in the highway scenario.

The TARS protocol shows less average delay as compared to the DBSR and POVRP. TARS uses a traffic congestion avoidance algorithm which reduces the unnecessary messages caused by vehicular congested traffic. The RSU only transmits warning messages and vehicles exchange safety messages according to the present traffic condition (i.e., vehicle velocity and the distance between each vehicle to the other vehicle). TARS overcomes the condition in which vehicles move at high speed and broadcast lots of messages, where there are a large number of vehicles causing congestion due to message dissemination. Moreover, after detecting congestion, the RSU transmits warning messages at the exact time for the upcoming vehicle to select an alternate route in order to prevent congestion and avoid accidents, the upcoming vehicle makes more intelligent decisions and selects a less congested route while applying a basic safety message strategy (vehicles share their velocity and vehicle-ID with other vehicles) to follow the specified path. In this way, the TARS protocol provides less delay in order to transmit warning messages, as a result, driver safety is increased, as shown in Figure 12.

### 5.3. Message Delivery Ratio (MDR)

**Figure 13:**
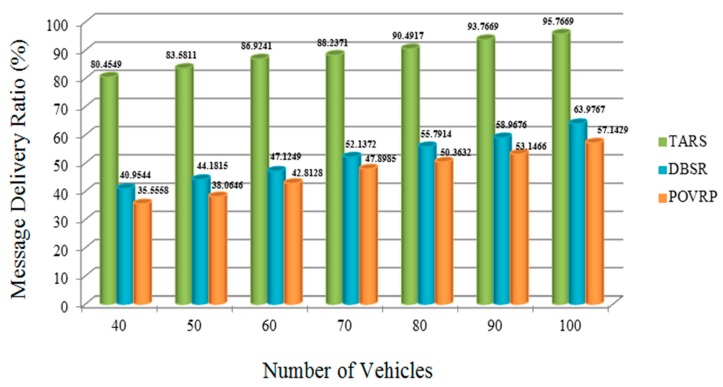
Message Delivery Ratio w.r.t. the Varied Vehicle Density in the highway scenario.

The TARS protocol shows a high MDR, as compared to the DBSR and POVRP with different numbers of vehicles. When the number of vehicles increases, then the rate of messages also increases. If the RSU and vehicles both transmit the messages, then heavy congestion in the channel will result, the frequency of transmitting messages gets reduced, and the messages cannot be delivered to the neighboring vehicles. Moreover, due to the unsuccessful reception of messages, there are a lot of chances of the data packet loss and due to this packet loss some safety messages also experience loss and cannot reach the specified destination. In order to overcome this challenge, the TARS protocol plays a very important role and shows high PDR, meaning the protocol builds a reliable connection between the two vehicles as well as the connection between the RSU and vehicle, with minimum packet and message loss [39,40,41,42,43]. The RSU transmits the warning message up to one hop neighbor (which lies in its transmission range) and the direction based forwarding mechanism minimizes the packet loss chances, which also helps to improve the packet delivery ratio [44]. The protocol shows the successful packet transmission of warnings as well as safety messages with maximum reach, and the ability to improve driving safety through more reliable delivery of BSMs in VANETs, as shown in Figure 13. Furthermore, the MDR achieves successful transmission to the specified destination; for the MDR required, 7, 8, and 9 out of 10 messages per second must be calculated and successfully received from the other vehicles or RSU.

### 5.4. Confidence Interval in Terms of Message Delivery Ratio

In order to calculate the confidence interval, the highway scenario is evaluated and analyzed. The geographical (highway) trace file was retrieved from SUMO in order to achieve the real world scenario. Simulation was conducted in NS-2, neighboring vehicles with 40–200 vehicles travelling random routes with the help of a random mobility model. For each configuration, 50 trials were executed for 500 s and the simulation has been conducted with different random seeds; as a result, MDR results were achieved in the transmission range of 30 m–2100 m that are assumed to be normally distributed.

**Figure 14:**
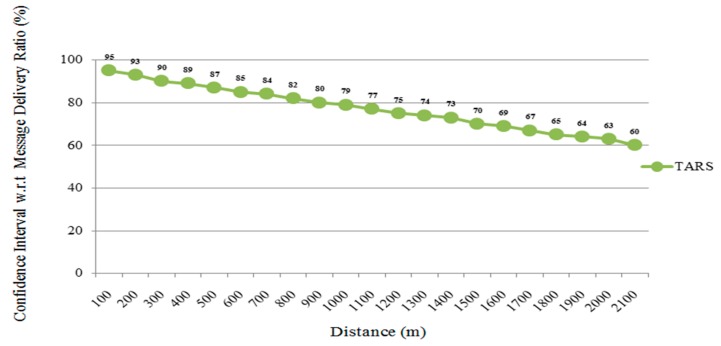
Confidence Interval w.r.t Message Delivery Ratio in the highway scenario.

The MDR showed the average probability of receiving a single message with the confidence interval. Figure 14 shows the 95% confidence interval in the highway scenario. The MDR shows the coverage of message dissemination, when the source vehicle transmits the message and the simulation computes the increase of message coverage with time. If the message coverage greatly increases in a short interval, then it means the proposed protocol achieves high message dissemination performance. TARS shows a 95% confidence interval as shown in Figure 14. In the highway scenario, the distance between vehicles is larger as compared to the dense areas. Therefore, by using a predefined threshold value, the MDR requires that 6, 8, and 9 out 10 messages per second are received and are calculated to have an approximately 75%, 88%, and 95% confidence interval, respectively, in a case when the distances between the vehicles are smaller [45]. However, when the number of vehicles is increased then the number of messages also increases as well as the communication becoming difficult in cases when the distance between the vehicles is greater. So, there are a lot of chances that the single message does not reach the specified destination and the rate of message loss becomes greater when the number of vehicles is increasing [45].

### 5.5. Message Loss Ratio (MLR)

**Figure 15:**
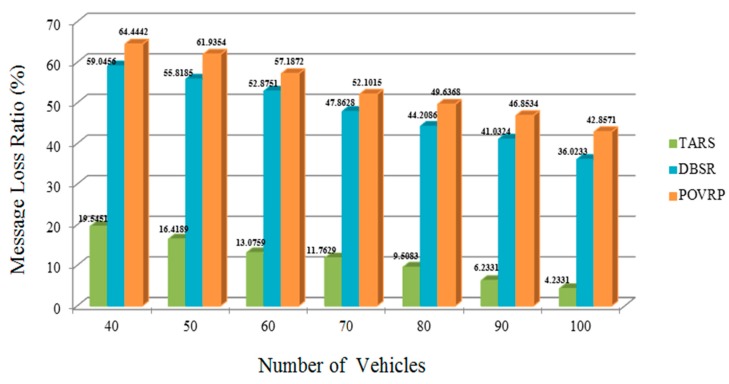
Message Loss Ratio w.r.t. the Varied Vehicle Density in the highway scenario.

We evaluated and analyzed the loss warning messages or safety messages due to an unstable link connection. The TARS protocol transmits messages successfully at the specified destination. In this way TARS shows less loss ratio as compared to other similar studies. The warning and beacon safety message must be delivered to each neighboring vehicle without delay. A single delay or loss of any packet could result in loss of life. However, if the number of vehicles increases then the chances of lost packets also increase, as result causing instability of the network. Therefore, the TARS protocol shows less loss packets as compared to DBSR and POVRP. It means the proposed protocol provides the stability of the network and more packets or messages are delivered up to the specified destination. So, TARS provides a better and reliable routing scheme as per shown in Figure 15.

### 5.6. Discussion

We have highlighted the issue of road safety in Section 1. In Section 2, we summarized and discussed the literature review. In Section 3, we proposed a strategy named as the Traffic Accidents Reduction Strategy (TARS) that aimed to avoid vehicle collision and re-route road traffic to another route so that the traffic keeps flowing. In Section 4, we analyzed and evaluated the performance of our proposed protocol scheme. TARS showed a better performance in Message Delivery Ratio (MDR), Message Loss Ratio (MLR), Average Delay, and Basic Safety Message Rate. In the end, we concluded that all of the step-by-step work in scientific steps, theories, mathematical calculation and performance results evaluation and discussion were verified by supporting explanations. In Section 5, we discussed the experimental results and compared the performance of TARS protocol with the other similar studies of routing protocols like DBSR and POVRP [37,38].

## 6. Conclusions

Our research paper has highlighted the issue of road safety. Road hazards have drastically increased with the increase in automobiles over the last ten years. Many efforts have been made to avoid such severe conditions. However, these efforts are restricted in providing a holistic solution. The proposed protocol, TARS, forecasts the probability of the occurrence of an accident in advance before it occurs. It also re-routes vehicle traffic to prevent traffic jams on the road that may cause accidents. TARS is aimed at maintaining the traffic flow efficiently. It also assists drivers in reaching a destination on time. Moreover, TARS reduces the safety messages broadcasting in order to avoid a broadcasting storm and network congestion and minimizes the delay in re-routing of traffic to other available paths. Our results demonstrated that TARS had a good performance in Message Delivery Ratio, Message Loss Ratio, Average Delay, and the Basic Safety Message Rate. In future, our goal is to propose a mathematical model and perform further simulations on performance metrics that are associated with TARS in an urban scenario. Our results and discussion showed a successful demonstration of the TARS proposed scheme as compared to other similar studies.

## Figures and Tables

**Table 1:** Literature review of traffic accident prevention and avoidance schemes based on Safety/Warning Messages.

Authors	Proposed Scheme Name	Proposed Scheme Methodology	Issues Identified	Benefits	Scalability Issue	Simulation Tool
Garcia-Lozano et al. [16]	Warning Message routing scheme	The warning message scheme has been introduced for low priority messages in order to achieve efficient bandwidth utilization. The scheme achieves the best high and low priority message dissemination in drastic weather conditions	Drivers’ reaction time when some situations occur. Delay in message broadcasting	Less delay efficient utilization of the bandwidth	Yes	NS-2.34
Gokulakrishnan et al. [17]	Road Accident Prevention (RAP).	When RSU detects any unusual activity, broadcast EM message to all those vehicles which are lying in the range of the RSU. EW message must be sent at the exact period to prevent road accidents.	Timeliness of alert messages Delay in message broadcasting	Less delay	No	NS-2.0
Salman Dawood et al. [18]	Efficient Emergency Message Broadcasting (EEMB)	The affected vehicle selects the best forwarder and broadcasts the emergency message. The forwarder vehicle rebroadcasts the emergency message to the RSU. This procedure continues until the emergency message covers the whole risk zone. The proposed scheme overcome the problem of network fragmentation by using store-carry and forward.	The Collision probability to evaluate the communication in VANETs. Timeliness of alert messages	Less message overhead	No	MATLAB, R2011b Version
Benslimane et al. [19]	Optimized Dissemination of Alarm Message (ODAM)	Vehicles which have the alarm message must broadcast the message until it selects another forwarder vehicle.	Detection of the Traffic Congestion Flooding	Less overhead Less end-to-end delay	No	MATLAB
Roy et al. [20]	Traffic congestion detection and avoidance scheme	An RSU broadcasts the emergency message to the other RSUs which lies in its range. This prevents the duplication of an emergency message. These messages help the driver to select another route to avoid accidents.	Distribution of the capacity of the bandwidth. Broadcasting storm problem Detection of Traffic congestion.	High throughput Less end-to-end delay High packet delivery ratio	No	NS-2.0

**Table 2:** Literature review of traffic accident prevention and avoidance schemes based on Routing Schemes.

Authors	Proposed Scheme Name	Proposed Scheme Methodology	Issues Identified	Benefits	Scalability Issue	Simulation Tool
Rajesh-Kumar et al. [21]	Distance Based routing scheme	The scheme obtains the vehicle location and calculates the distance of the vehicle that is near to the intersection. After calculating the location, the vehicle with the shortest distance to the intersection will send an alert to the other vehicles about its distance and location. All other vehicles will be alerted about the upcoming vehicle and are protected from the traffic accident	Possibilities presented at the intersection. Timeliness of alert messages	Less traffic congestion Maximum Packet Delivery Ratio.	Yes	NS-2.34
Nzouonta et al. [24]	Spatio-Temporal Emergency Information Dissemination (STEID)	The routing scheme satisfies both temporal and spatial reliability by guaranteeing the delivery of an alert message in a short interval The message passes to all the vehicles that are passing through the zone during the lifetime of an emergency.	Delay in message broadcasting	Maximum delivery ratio of alert messages Less traffic load Less delay w.r.t message reception rate	No	NS-2.29
Devdhara et al. [27,28]	Inter-Vehicle Collision (IVC) scheme	All vehicles in the cluster broadcast secure the message to give further information to the other vehicles. A traffic accident which lies in the same cluster is detected and information about the accident is sent to the other vehicles in the cluster.	Simple Flooding	Cluster size Driver reaction time Transmission range	No	SUMO OMNET++ VEINS Clustering Algorithm Techniques
Bhumkar et al. [29]	Driver fatigue detection scheme	The proposed scheme used real time sensors to detect the driver’s fatigue immediately. Upon detection, it warns through a notification message and then turns the ignition off. The scheme performs very well on human behavior like drinking alcohol.	Human behavior causes traffic accidents	Driver reaction time Transmission range	No	ARM7 MQ-3 gas sensor GPS Smart Receiver Google Map API Visual Basic (VB)
Nzouonta et al. [30]	Road Based Vehicular Traffic (RBVT) routing scheme	The proposed routing scheme used the geographical forwarding scheme in order to transmit interest packets between road intersections on the route. The proposed scheme performs very well on the intersections. AODV protocol is used to discover the route.	Possibilities use the presented intersection. The collision probability is used to evaluate the communication in VANETs.	Average packet delivery ratio High Average delay due to its traffic overhead.	No	NS-2.30 SUMO DCF standard with IEEE 802.11p
Manoj et al. [31]	Traffic congestion detection and avoidance scheme	After the detection of traffic congestion, the drivers of the vehicles provide magnitude and location of the vehicle. The affected vehicle, which is involved in the traffic accident, broadcasts the warning message to the RSU. The RSU further rebroadcasts the warning message to make a decision on an alternate route.	Distribution of the capacity of the bandwidth. Detection of the Traffic Congestion.	Efficient bandwidth utilization Minimum message overhead	No	Net Beans IDE 7.0 Java
Khatri et al. [32]	Traffic congestion detection and avoidance scheme	The best forwarder vehicle collects the congestion message. It checks if the area is congested or not. If not, then the vehicle stores this message in its own memory. Otherwise, it simply shares the congestion location with the other vehicles [33].	Distribution of the capacity of the bandwidth. Detection of the Traffic Congestion.	Less transmission overhead Efficient utilization of the bandwidth	No	NS-2.0 SUMO

**Table 3:** Properties of the TARS simulation parameters.

Network Simulator	NS-2.35
Transmission Range RSU	400 m
Waiting Time	10 s
Total No. of Vehicles	40, 50, 60, 70, 80, 90, 100
Packet Size	1024 bytes
MAC Layer	IEEE 802.11p
Total number of Lanes on highway	4
Velocity Threshold	40 m/h
